# Supplementary material for: Group‐Patch Joint Compression: Compressing Dynamic B0 and Static RF Spatial Modulations Across k‐Space Subregion Groups for Highly Accelerated MRI
Source: Magn Reson Med. 2026 Jul 23;96(5):2106–26. doi: 10.1002/mrm.70439 (PMC13527271; doi:10.1002/mrm.70439)
Supplement: Supplementary file 1 [file MRM-96-2106-s001.pdf]

Supporting Information for

Group-Patch Joint Compression: Compressing Dynamic B_0 and Static RF Spatial Modulations Across k-Space Subregion Groups for Highly Accelerated MRI

Rui Tian[1]*, Klaus Scheffler[1,2]

[1]High-Field MR center, Max Planck Institute for Biological Cybernetics, Tübingen, Germany

[2]Department for Biomedical Magnetic Resonance, University of Tübingen, Tübingen, Germany

*Corresponding author: rui.tian@tuebingen.mpg.de

This Supporting Information provides additional technical details about the group-patch joint compression approach, and comparisons with the conventional RF receiver array compression. Technical information about the image acceleration by local B_0 coils-SENSE and Wave-CAIPI/CS-Wave are also shown.

1 Investigating data compression using ex-vivo phantom scans

In this section, more technical details of data compression and image reconstruction are presented, based on retrospectively undersampled ex-vivo phantom scans without in-vivo motions.

The Figure 7 of the manuscript can be summarized by the Table S1 as below, to help understanding:

Compression	Encoding map	k-space “stamp”	Power function/TR	G-map
None	Physical sensitivity	GRAPPA kernel	Single trough	mean/max: 1.1/1.5
4x RF-only	Slightly altered	Slightly dispersed	Single trough	Worse
4x joint	Strongly oscillatory	Extremely dispersed	Splits, Two troughs	Unchanged
8x joint	Strongly oscillatory	Extremely dispersed	Two troughs, larger approximation errors	Unchanged
12x joint	Oscillatory	Dispersed	Two closer troughs	Unchanged
24x joint	Similar to physical B_0 and B_1 maps	Slightly dispersed	Slightly wider single trough	Slightly worse
72x joint	Only most essential patterns retained	Small/compact	Narrower single trough, larger approximation errors	L_2 recon. impossible

Table S1. Summary of transitioning characteristics of different compression types and factors in Figure 7.

The 2D and 3D ex-vivo (without motions) reconstructions using the reconstruction algorithm #2 (FISTA, compatible for random sampling masks) were investigated as shown in Figure S1-S2, which highlights variations in reconstruction quality and computational speeds (i.e., computing

compression matrices, applying compression, reconstructing images) across different compression types and factors. This comparison excludes the reconstruction algorithm #1 (only compatible with regular/CAIPI sampling masks), focusing solely on the dependency of reconstruction performance on conventional RF-only compression versus our proposed joint compression.

1.1 2D compression, reconstruction algorithm #2

In Figure S1, a 2D FLASH ex-vivo scan accelerated by rapid B_0 modulations and multiple RF receivers was reconstructed with various compression factors for the readout forward model. We used a generic FISTA-based code (i.e., reconstruction algorithm #2) compatible with both least-squares (i.e., disabling nonlinear regularizations) and compressed sensing reconstruction to compare reconstruction speed and accuracy, with varying compression types and factors.

Figure S1A shows multiple sampling patterns, including fully sampling, 6x regular undersampling of phase-encoded steps, and the 6x undersampling pattern with additional 5 central k-space calibration lines (i.e., equivalent ACS lines with B_0 modulations).

The least-squares reconstruction with 6x-undersampling shows noise amplification as expected. Based on the visible image quality, and the quantitative metrics (i.e., NRMSE and SSIM), the comparison of regular 6x-undersampled reconstruction quality is (“>” means “better than”):

No compression > joint compression 24x > RF compression 4x

The computation time of joint compression is about 1.2s longer than RF compression, due to larger size of the encoding matrices, although this might start before the MRI scan finishes. However, the reconstruction time for the jointly compressed forward model is 3.4s less than the one by RF compression.

The compressed sensing reconstruction has even better reconstruction quality based on the NRMSE and SSIM, mainly due to nonlinear regularizations. The 24x joint compression makes the CPU reconstruction time within only 1.4s.

Overall, the joint-compression is much more efficient than the RF-only compression, although the 2D reconstruction with a pixel size of 180 x 180 doesn't cost that much time.

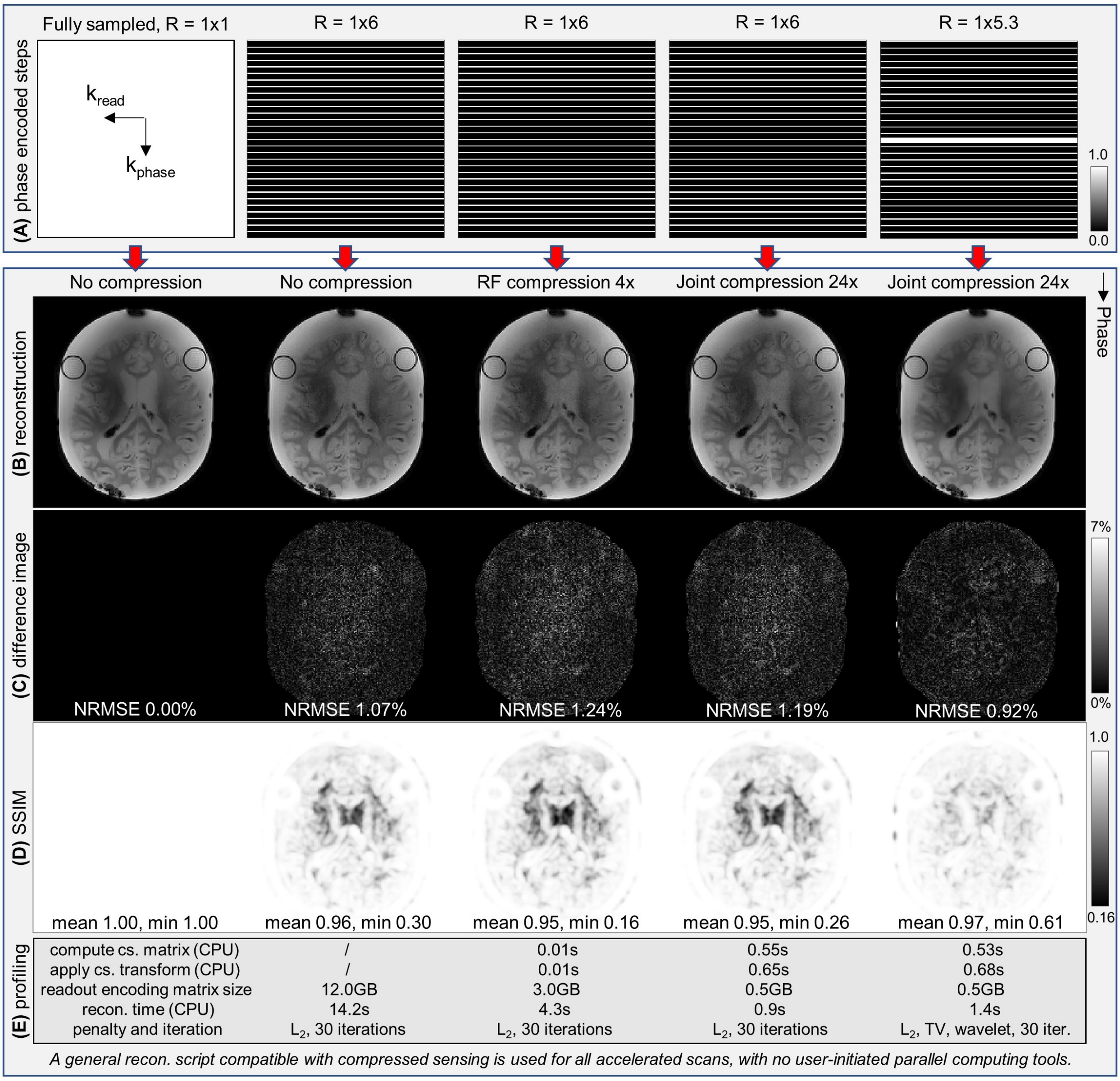

Figure S1. The reconstruction of a 2D scan accelerated by rapid B_0 modulation and multiple RF receives, with no compression, RF array compression and joint compression. A. k-space undersampling mask. B. reconstructed image. C.difference image between the reconstruction and the reference image without compression and undersampling. D. SSIM comparing the reconstruction and the reference image without compression and undersampling. E. computational profiling. With no undersampling and no compression, the reference image sets a baseline of 0.00% NRMSE and 1.00 SSIM.

1.2 3D compression, reconstruction algorithm #2

In Figure S2, a 3D FLASH ex-vivo scan accelerated by rapid B_0 modulations and multiple RF receivers was reconstructed with different compression factors for the readout forward model.

Figure S2A shows multiple 2D sampling patterns for the two phase-encoding dimensions, including fully sampling, regular undersampling using a 4x3 CAIPI pattern, VD-PD sampling patterns with a 10x10 fully sampled k-space center (i.e., equivalent ACS lines with rapid B_0 modulations).

The least-square reconstructions with 4x3 CAIPI undersampling exhibit noise amplifications as expected. However, based on the NRMSE and the SSIM, the reconstruction quality shows quantitative comparison, for both cases without and with nonlinear regularization (“>” means “better than”):

Joint compression 18x > RF compression 3.2x

The computational duration for jointly compressing larger matrices takes more time (here, ~47s) than RF only compression (~1s). However, this is negligible compared to the reconstruction time reduction due to jointly compressed readout forward model, dropping from 691.4s to 112.2s for least-square reconstruction, and decreased from 716.9s to 176.6s for compressed sensing reconstruction. This is because, correspondingly, the readout encoding matrix size was reduced from 298.6GB to 53.1GB.

Overall, the proposed joint compression for 3D scans is not only more efficient than RF array compression in terms of signal encoding, but also drastically lowers the computational demands. This ensures that the data expansion from highly-oversampled readout does not inappropriately exceed the undersampled data amounts, and thus, would not incur unreasonable computational burden especially for advanced iterative reconstruction using e.g., compressed sensing.

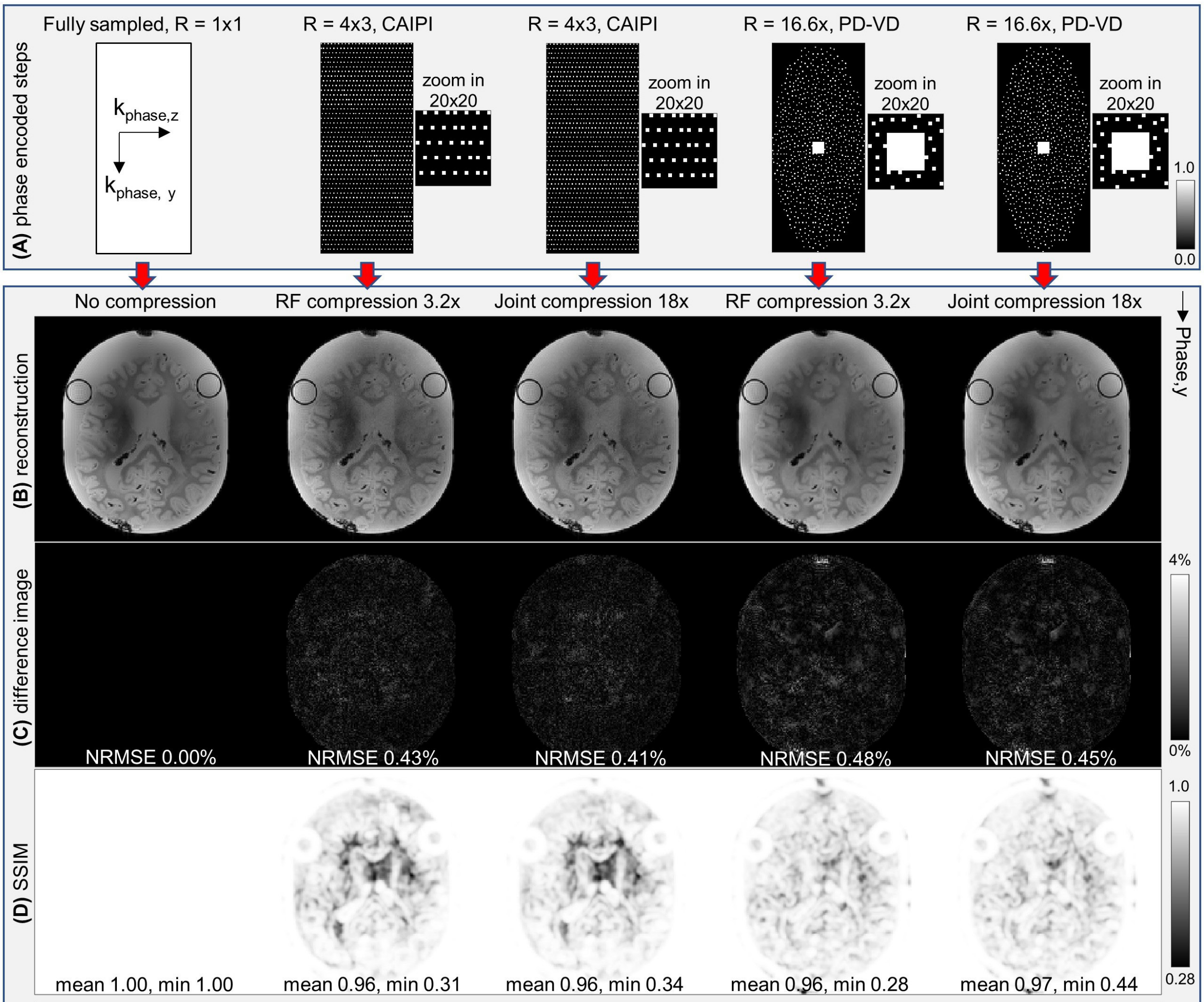

compute cs. matrix (CPU)	0.6s	10.2s	0.5s	10.3s
apply cs. transform (CPU)	0.3s	37.0s	0.3s	37.1s
readout encoding matrix size	298.6GB	53.1GB	298.6GB	53.1GB
recon. time (CPU)	691.4s	112.2s	716.9s	176.6s
penalty and iteration	L_2, 70 iterations	L_2, 70 iterations	L_2, TV, wavelet, 70 iter.	L_2, TV, wavelet, 70 iter.

Figure S2. The reconstruction of a 3D scan accelerated by rapid B_0 modulation and multiple RF receives, with no compression, RF array compression and joint compression. A. k-space undersampling mask. B. reconstructed image. C.difference image between the reconstruction and the reference image without compression and undersampling. D. SSIM comparing the reconstruction and the reference image without compression and undersampling. E. computational profiling. With no undersampling and no compression, the reference image sets a baseline of 0.00% NRMSE and 1.0 SSIM.

1.3 3D compression, reconstruction algorithm #1 and #2

Furthermore, reconstruction times for the 3D ex-vivo FLASH scan with rapid B_0 modulations using both reconstruction algorithms (#1 and #2) are listed in the Table S2. This computational profiling helps guide the choice of reconstruction algorithms for different applications, such as varying sampling mask types or regularization strategies. In all cases of such matrix size, our joint

compression technique substantially speeds up the reconstruction, compared to RF-only compression.

Hardware specification	The k-space undersampling pattern	3D recon. algorithms MATLAB, single pixel size 180x180x80 readout os. x8	Compress type	Read encoding matrix size	Time	Iteration
HPC cluster 2xAMD EPYC 7452 2.35GHz 64 cores 2 hyper-threads/core 1T MEM	R = 4x3 regular CAIPI	read: decoupled, LSQR phase: 2D-FFT CPU	RF 3.2x	0.2GB	690s	auto
			Joint 18x	0.04GB	170s	auto
		read: decoupled, LSQR phase: 2D-FFT CPU, “parfor” used	RF 3.2x	0.2GB	79s	auto
			Joint 18x	0.04GB	19s	auto
		FISTA, L_2 only read: full model phase: 2D-FFT CPU	RF 3.2x	298.6GB	691s	70
			Joint 18x	53.1GB	112s	70
	R = 16.6x VD. Poisson-Disc	FISTA, L_2+TV+wavelet read: full model phase: 2D-FFT CPU	RF 3.2x	298.6GB	717s	70
			Joint 18x	53.1GB	177s	70

Table S2. Computational profiling of different reconstruction algorithms. The dataset is a 3D GRE ex vivo scan accelerated by rapid B_0 field modulations and 32 RF receivers, given an image pixel size of 180 × 180 × 80 and a k-space readout oversampling factor of 8. The “time” considers only the reconstruction time duration of the iterative algorithms, excluding the time for loading, saving and preparing the data and encoding matrix which were fed into the iterative reconstruction algorithms. Note the wavelet function in MATLAB was internally double-precision.

For decoupled phase-encoding pixels with regular 4x3 CAIPI undersampling pattern, the least-squares reconstruction was reduced from 690s using 3.2x RF compression, to 170s using 18x joint compression. Parallelizing the inversion of these decoupled readout forward models further reduced reconstruction time to 79s or 19s, respectively. For a generic script of FISTA (reconstruction algorithm #2) with an option to disable the nonlinear regularizations for comparing the least-square

only computational time, the reduction factor of reconstruction time due to joint compression (112s) is similar to the reconstruction algorithm #1 that decouples sets of overlapped phase-encoding pixels without parallel CPUs (170s). The reconstruction algorithm #1 is not implemented with total variation and wavelet regularization. For compressed-sensing reconstruction with a Poisson-Disc undersampling mask, the FISTA took only 177s for reconstruction with jointly compressed readout forward model, substantially faster than the one based on RF array compression which took 717s.

In both cases, 18x joint compression delivered an additional 4x to 6x speed up over conventional RF-only compression, primarily because of a 5.6x smaller readout encoding matrix, without decreased encoding efficiency due to compression.

2 RF-only vs. B_0-RF compression in-vivo 2D scans at 9.4T

In Figure S3, with reasonable compression factors, reconstructions of 2D in-vivo FLASH with local B_0 coils modulations are shown, comparing the RF-only compression and the proposed group-patch joint compression.

Given 7x undersampling, the least-squares reconstruction (using the reconstruction algorithm #1) quality based on both 20x joint compression and 4x RF compression is nearly identical to the one without compressions. They have only slight noise amplifications relative to the non-compressed and fully-sampled least-squares reconstruction. With compressed-sensing reconstruction (using the reconstruction algorithm #2), the image quality based on both compression algorithms is nearly identical to the non-compressed, fully-sampled least-squares reconstruction. Here, the local B_0 modulations provided sampling incoherence for 2D compressed-sensing, where the 1D uniform undersampling pattern was used.

For joint compression, its compression time alone was slightly longer than RF compression, but its total time for compression and reconstruction became less, especially in compressed-sensing reconstructions (i.e., 3x faster).

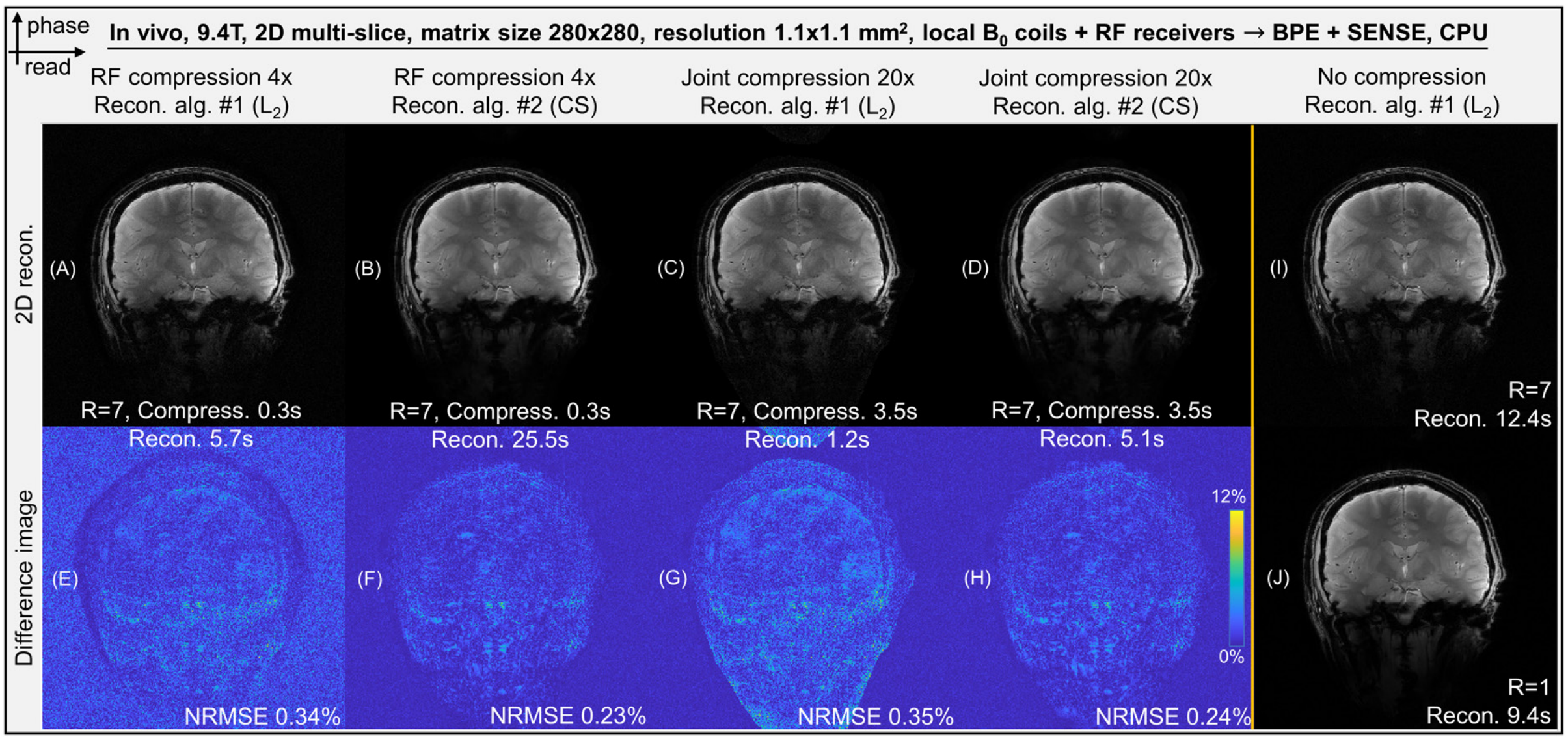

Figure S3. 2D least-squares and compressed-sensing reconstructions with reasonable compression factors for RF-only and joint compression algorithms at 9.4T. The rapid field modulations were provided by local B_0 coils. The varaible-density Poisson-disc (VD-PD) mask was generated by BART. (A-D): L_2 and CS reconstructions with 7x undersampling: 4x RF compression for L_2 reconstruction, 4x RF compression for compressed-sensing reconstruction, 20x joint compression for L_2 reconstruction, 20x joint compression for compressed-sensing reconstruction. (E-H): Different images relative to the non-compressed, fully-sampled image. (I) non-compressed, 7x-undersampled L_2 reconstruction. (J) non-compressed, fully-sampled L_2 reconstruction. Note, (J) was also reconstructed from the same local B_0 modulated scan, and thus, the difference maps here cannot fully reflect field-calibration artifacts, nor inter-scans subject motions. The compression and reconstruction time are shown. In L_2 reconstructions, MATLAB tool “parfor” was used. Here, the CS reconstruction incorporates additional wavelet and total variation regularizations, while still using a 1D regular undersampling mask for 2D reconstruction.

3 Comparing RF-only and joint compression under identical compression factors

In Figure S4, using identical compression factors, reconstructions using the RF-only and the proposed joint compression techniques are compared, for 2D and 3D in-vivo FLASH with local B_0 coil modulations at 9.4T.

In 2D FLASH with 7x undersampling, 16x RF compression (2 virtual coils) leads to strong artifacts. However, joint compression by both 16x and 8x have minimal artifacts. Note even moderate (8x) RF-only compression slightly degrades encoding efficiency than the joint compression, indicated by difference maps in the same scale.

In 3D FLASH with 4x3 undersampling, both 32x and 16x RF-only compression (1 and 2 virtual receiver coils) have strong artifacts. The 32x joint compression has only slight fold-over artifacts, which are removed in the 16x joint compression.

Note that, especially in 3D, joint compression takes noticeably longer than conventional RF array compression, because the SVD operates on a much larger signal space that additionally includes the oversampled readout points. Nevertheless, while compression can begin before the scan completes, the total computational time and peak memory for 2D and most 3D scans with joint compressions can still be substantially reduced compared to a realistic RF-only compression, as shown in manuscript.

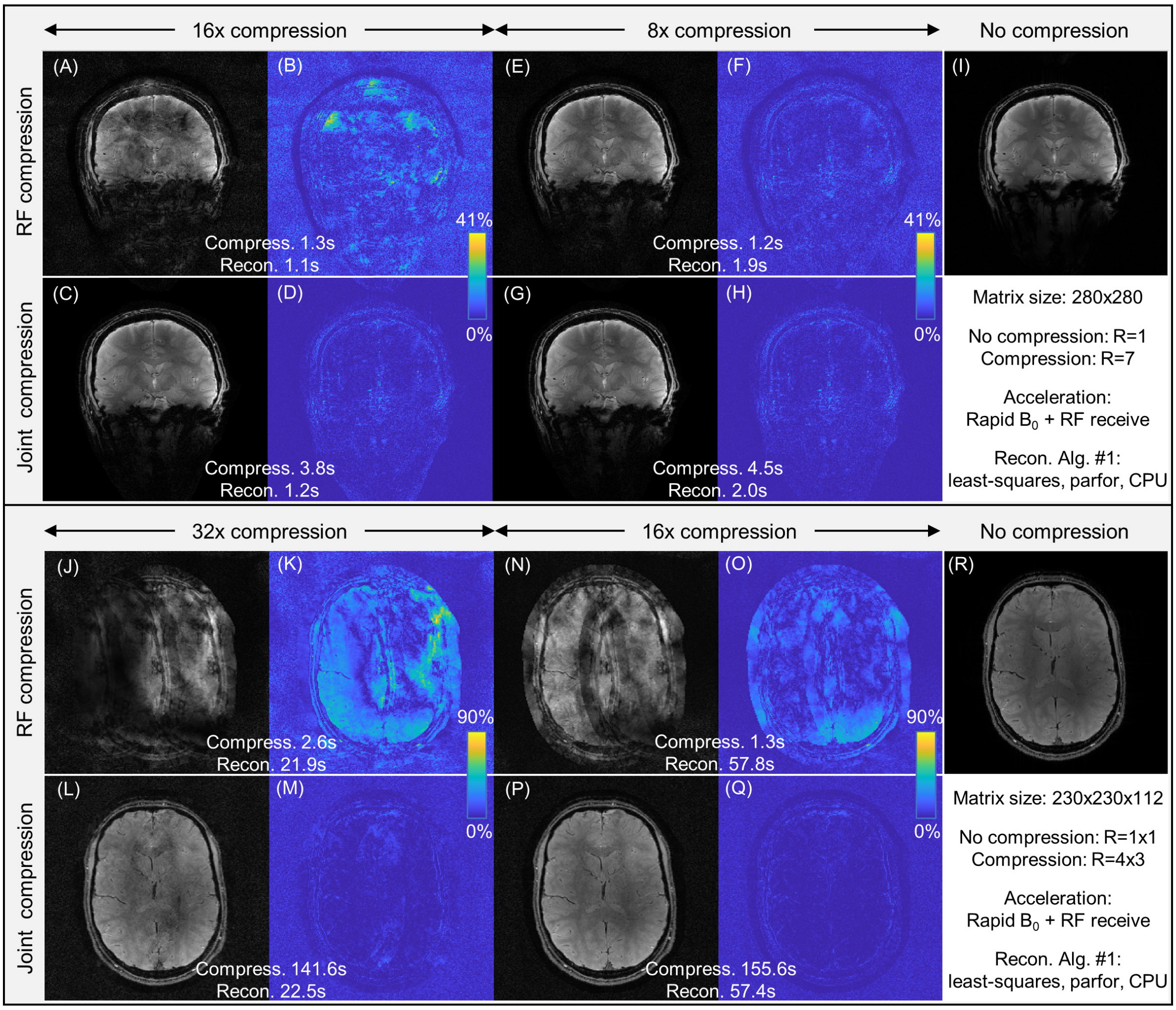

Figure S4. 2D and 3D least-squares reconstructions with same compression factors at 9.4 T, comparing RF-only and joint compression algorithms. The rapid field modulations were provided by local B_0 coils. (A–D): 16× compression for a 2D scan

with 7× undersampling. (E–H): 8× compression for a 2D scan with 7× undersampling. (I): reference fully-sampled 2D image without local B_0 modulations and compressions. (J–M): 32× compression for a 3D scan with 4 × 3 undersampling. (N–Q): 16× compression for a 3D scan with 4 × 3 undersampling. (R): reference fully-sampled 3D image without local B_0 modulations and compressions. Next to the reconstructions, the difference images were calculated relative to the reference FLASH images, which should be free of any artifacts caused by local B_0 coil or compression errors, but might contain subject motions relative to the field-modulated scans. The compression and reconstruction time are shown. Hybrid-space least-squares reconstruction with MATLAB tool "parfor" was used.

4 Increased encoding efficiency by rapid B_0 field modulations

This section provides supplementary information on encoding efficiency for in-vivo scans shown in Figure 8-9 of the manuscript, and Figure S3 in Supporting Information. Encoding efficiency for least-squares reconstruction can be accessed in either image-space or k-space. The image-space G-factor maps quantify pixel-wise noise amplification factors. The k-space cardinal function maps based on the RKHS formalism are the Frobenius norm of the k-space interpolation weights, and represent k-space noise amplification. The k-space power function maps based on the RKHS formalism characterize local k-space approximation error and represent the effective k-space sampling coverage. Here, we analyze encoding efficiency for the in-vivo scans in the manuscript, to highlight the acceleration potential in data acquisitions (note, not reconstruction speed) enabled by the rapid B_0 modulations, generated by either local B_0 coils or scanner's linear gradients. The encoding efficiency maps are computed from non-compressed encoding matrices. Note that, for G-map statistics, the boxplot in MATLAB is utilized. The central mark indicates the median, and the bottom and top edges of the box indicate the 25th and 75th percentiles, respectively. The whiskers extend to the most extreme data points not considered outliers, and the outliers are plotted individually using the red "+" marker symbol. These outlier G-map values can originate from those regions (e.g., background, skull) where B_0 field maps are difficult to calibrate, and thus, may not be meaningful to interpret the overall encoding efficiency. However, they are still plotted to remind the presence of these outliers.

As in Figure S5, for 2D coronal scans in Figure S3, we evaluate the B_0 field, the gradient field, the G-maps, the cardinal and power function maps, and compare reconstructions between SENSE-only acceleration and joint acceleration with rapid B_0 modulations and SENSE. The additional rapid B_0 modulations, when combined with multiple RF receivers, achieves a 7x acceleration factor for 2D FLASH, whereas conventional SENSE-only acceleration suffers from strong image artifacts.

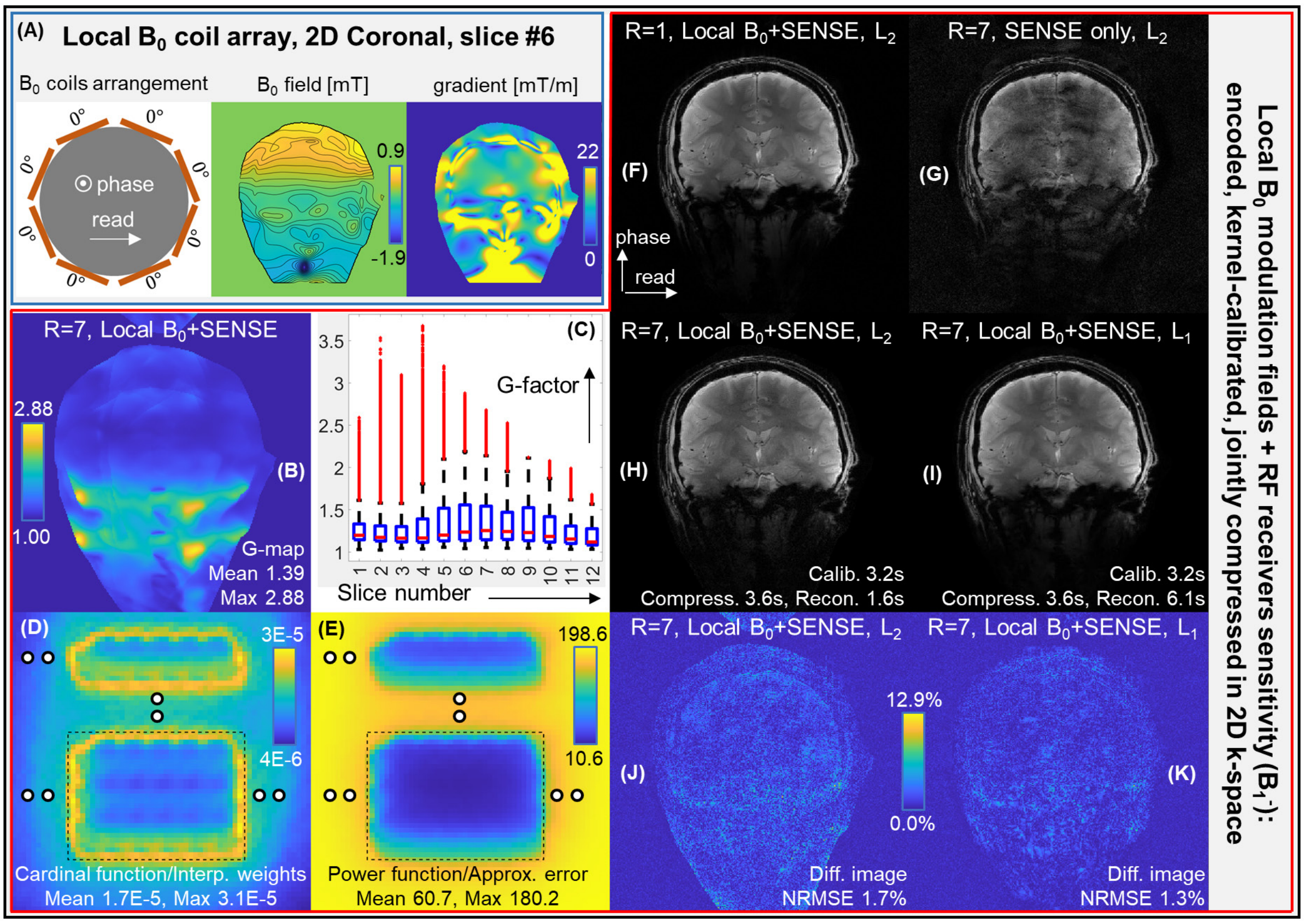

Figure S5. Supporting Information for Figure S3: Accelerated data sampling enabled by the rapid B_0 modulations generated by the local B_0 coil array at 9.4 T. (A) The phase offsets in local B_0 coils, the magnetic field distribution generated at nearly-peak current, and the estimated gradient strength distribution (Euclidean-norm in two orthogonal dimensions) are shown. (B–E) The G-map, the G-factor boxplot for each of 12 slices, the k-space cardinal and power function maps are shown, which indicate sufficient sampling to reach 7× joint acceleration factor by B_0 modulations and SENSE. (F–K) The reference fully-sampled image with B_0 and SENSE reconstruction is successfully reconstructed. The L_2 reconstruction of 7× undersampled k-space has only subtle noise amplifications without noticeable residue fold-over artifacts. Its L_1 reconstruction further improves the image quality, as also shown in their difference images, and is very close to the reference image. In comparison, the SENSE-only acceleration has severe artifacts. Here, the CS L_1 reconstruction incorporates additional wavelet and total variation regularizations, while still using a 1D regular undersampling mask for 2D reconstruction.

As in Figure S6, for 3D scans in Figure 8 of the manuscript, we show the k-space trajectory induced by rapid B_0 modulations, the k-space power function maps across readout timepoints, and the G-maps. Generally, the rapid B_0 modulations and multiple RF receivers yield reasonable encoding efficiency for such acceleration factors (i.e., 4x3, 4x2).

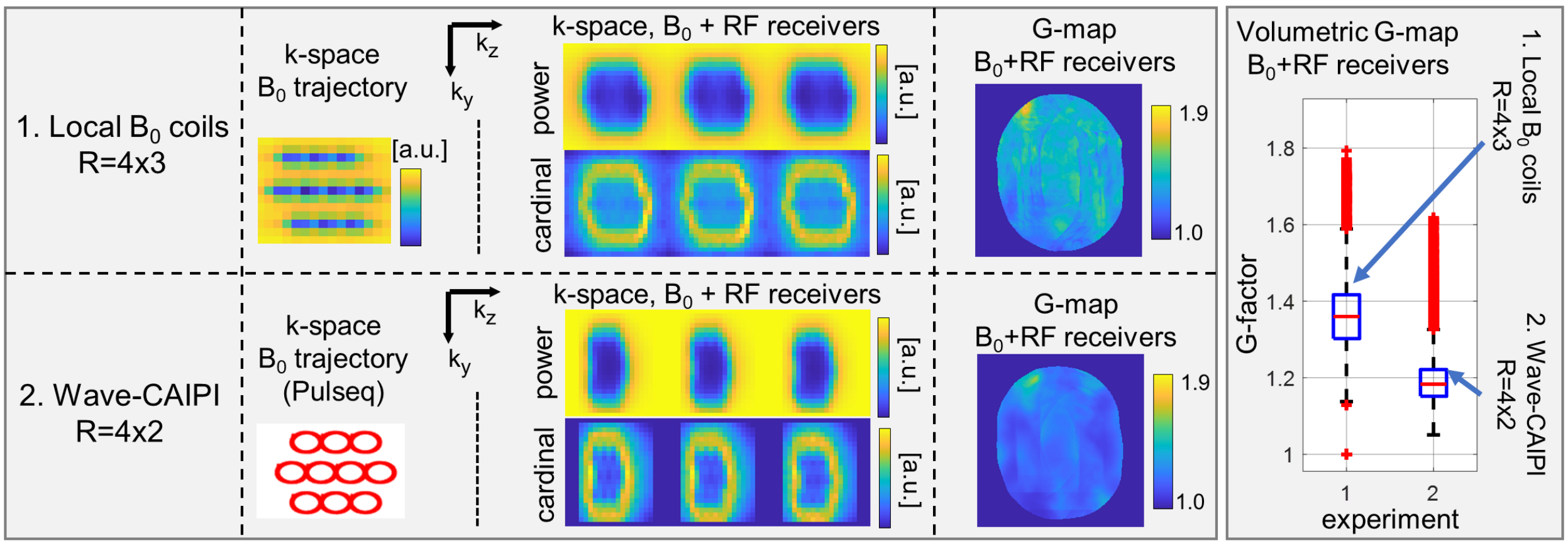

Figure S6. Supporting Information for Figure 8 of the manuscript: Accelerated data sampling enabled by the rapid B_0 modulations generated by the local B_0 coil array, or the scanner's phase-encoding gradients (i.e., Wave-CAIPI/CS-Wave), at 9.4 T. The k-space B_0 trajectory for local B_0 coils modulations is visualized using power function (effective k-space coverage) at a readout timepoint, considering a single RF receiver. The k-space B_0 trajectory for Wave-CAIPI is plotted from Pulseq built-in functions. The k-space power and cardinal function maps across three readout timepoints incorporate encoding by both rapid B_0 modulations and multiple RF receivers. The volumetric G-factors across the entire 3D volume are shown in the boxplots for the two different experiments, respectively. Note, the k-space efficiency maps based on the RKHS formalism are relative values and unitless. In both experiments, they show effective expansion of k-space coverage (thickened trajectories) by rapid B_0 modulations and multiple RF receivers. However, the detailed comparison between different experiments in the cardinal and power function maps are reserved for future study.

As in Figure S7, for 3D scans in Figure 9 of the manuscript, we show least-squares reconstruction, and the G-maps for the Wave-CAIPI scan. Here, the rapid B_0 modulations and multiple RF receivers provide reasonable encoding efficiency for the acceleration factor (i.e., 3x3) at 3T.

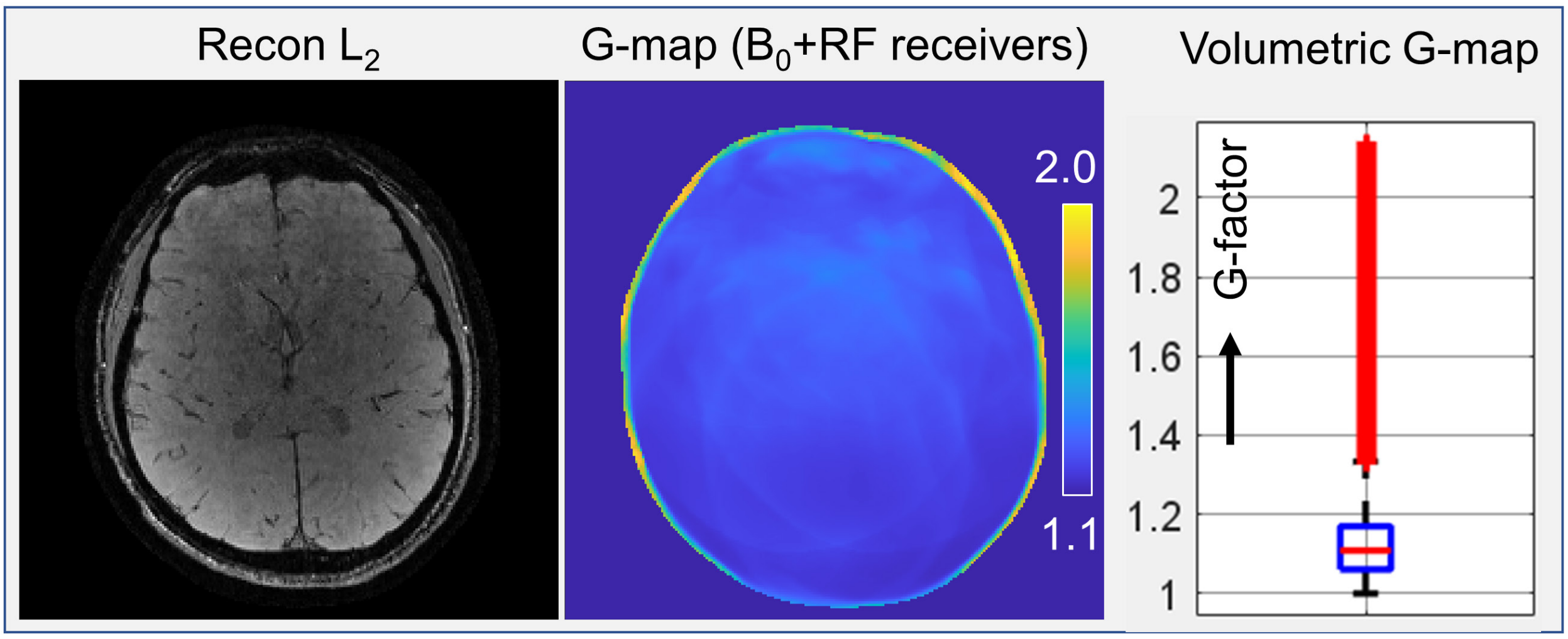

Figure S7. Supporting Information for Figure 9 of the manuscript: Accelerated data sampling enabled by the rapid B_0 modulations generated by the scanner's phase-encoding gradients (i.e., Wave-CAIPI/CS-Wave) at 3 T. Left: least-squares reconstruction. Middle: G-map for the corresponding slice position. Right: G-factor boxplot across the entire 3D volume.

5 Sequence protocols

In Figure 7, S1, the 2D multi-slice ex-vivo scan has a matrix-size of 180x180, corresponding to a resolution of 1.1mm x 1.1mm. The slice-thickness is 3 mm. The TE/TR is 4.91ms/30ms. The scanner's readout gradient bandwidth is 230Hz/Pixel. The 8-channel local B_0 coil array produced a nearly linear gradient with 7.41kHz/40A_{pk} currents per channel, with one sinusoidal period spanning 45 oversampled data points.

In Figure S2, the 3D ex-vivo scan has a matrix-size of 180x180x80, which includes a 33.3% slice oversampling and corresponds to a resolution of 1.1mm x 1.1mm x 1.0mm. The TE/TR is 4 ms/30 ms. The scanner's readout gradient bandwidth is 230 Hz/Pixel. The local B_0 coil array produces a nearly linear gradient along the z phase-encoded direction, and a quadratic field along the transverse phase-encoded direction, oscillating in 7.41kHz/40A_{pk} per channel, with one sinusoidal period spanning 45 oversampled data points.

In Figure S3, the 2D multi-slice in-vivo experiments was scanned with FOV 250x250mm^2, matrix-size 280x280, slice-thickness 3mm, readout-bandwidth 150Hz/Pixel, readout-duration 6.720ms, TE/TR 6.07ms/30ms. The B_0 coils currents were 7.41kHz/36A_{pk} per channel. One sinusoidal period spanned 45 oversampled data points.

In Figure 8 and S4, the in-vivo 3D experiments with local B_0 coils modulations was scanned with FOV 230x230x88mm^3, matrix-size 230x230x112 (slice-oversampling 27.3%), readout-bandwidth 180Hz/Pixel, readout-duration 5.520ms, TE/TR 6ms/30ms. The B_0 coils currents were 7.41kHz/36A_{pk} per channel. one sinusoidal period spanning 45 oversampled data points. In-vivo 3D Wave-CAIPI/CS-Wave (Pulseq) was designed similarly (i.e., identical FOV, matrix-size, readout-bandwidth, readout-duration, TE/TR. Nevertheless, the oscillating phase-encoding gradients (y-z) were (3.4-5.3) mT/m, 4.17kHz for wave-trajectory with 24 wave-cycles. One sinusoidal period spanned 80 oversampled data points.

In Figure 9, 3D Wave-CAIPI/CS-Wave (Pulseq) at 3T was scanned with 230x230x114mm^3, matrix-size 230x230x144 (slice-oversampling 26.3%), readout-bandwidth 180Hz/Pixel, readout-duration 5.520ms, TE/TR 20ms/35ms. The oscillating phase-encoding gradients (y-z) were (6.4-7.7) mT/m, 3.33kHz for wave-trajectory with 19 wave-cycles. One sinusoidal period spanned 100 oversampled data points.
